# Prevalence and Predictors of Storage of Unused Medicines among Households in Northwestern Ethiopia

**DOI:** 10.1155/2020/8703208

**Published:** 2020-03-26

**Authors:** Dawit Kumilachew Yimenu, Fitsum Sebsibe Teni, Awol Jemal Ebrahim

**Affiliations:** ^1^Department of Pharmaceutics and Social Pharmacy, School of Pharmacy, College of Medicine and Health Sciences, University of Gondar, P.O. Box 196, Gondar, Ethiopia; ^2^Department of Pharmaceutics and Social Pharmacy, School of Pharmacy, Addis Ababa University, Addis Ababa, Ethiopia; ^3^Department of Social and Administrative Pharmacy, School of Pharmacy, Jimma University, Jimma, Ethiopia

## Abstract

**Background:**

Unused medicines are those that are expired, discontinued, deteriorated, and/or not intended for any future use. The aim of this study was to assess the prevalence of unused medicines and predicting factors in households of Awi zone, Amhara regional state, Northwestern Ethiopia.

**Methods:**

A community-based cross-sectional study was conducted. A survey of unused medicines was conducted through interviews with representatives of households. The collected data were entered with Epi Data version 3.1 and exported to SPSS version 21 for analysis. Predictors of storage of unused medicines were assessed through binary and multivariable logistic regression methods. A confidence interval of 95% and a *P*-value of <0.05 were considered to declare statistical significance.

**Results:**

Of the total of 507 households surveyed, 70 (13.8%) were found to have unused medicines. These constituted twenty-eight types of unused medicines. Anti-infective medicines were the most commonly unused medicines, 58.9%. People who pay for medicines by themselves, those who lacked knowledge about medicines, and those who did not receive enough counseling about medicines they took were found to be 2.6, 4.8, and 3 times more likely to have unused medicines, respectively.

**Conclusion:**

A significant amount of unused medicines was present in the community. Strategies aimed at educating the public regarding the safe disposal of unused medicines and an organized method of collection and disposal of unused medicines in the community need to be introduced.

## 1. Introduction

It is a common practice for patients to be in possession of unused medicines. Unused medicines represent those medicines that are expired, discontinued, deteriorated, and/or not intended for any future use. According to the World Health Organization report, globally there is only 50 percent adherence to prescriptions in long term condition medicines [1].

It was reported that about £37.6 million ($62.4 million US) of drugs were estimated to be wasted each year in England by disposal programs delivered by community pharmacies [2]. A similar study conducted at University Of Gondar Specialized Teaching Hospital found that the majority of the study participants (89.1%) had unused medicines [3].

Antipain medicines, antimicrobials, medicines for chronic conditions, and antipsychotic medicines were found to be among the most commonly reported unused medicines [4]. A study in Nigeria has also reported analgesics, antibiotics, and nutrition/blood preparations as the most commonly unused medicines in the community [5].

Unpleasant side effects, symptoms being relieved, forgetfulness, dosage changes/change of treatment, unclear instructions on medicine use, progression of illness, medicines reaching the expiration date, intention not to waste them, lack of knowledge about the proper disposal method, or the death of some patients due to life-ending morbidities while on medicine are the most commonly reported reasons for nonutilization of medicines [3, 6–13]. The high cost of medicines, lack of disposal method, or the possibility of needing these medicines again in the future may also cause patients to keep medicines [14].

When unused medicines are allowed to accumulate at home, they will pose a risk to public health through poisoning and suicide and to the environment through poor disposal practices [15]. Sharing of unused medicines is one of the major reasons for the various adverse health outcomes such as unmonitored adverse drug events [16], complications in clinical diagnosis [16], drug resistance [17], and delay in care-seeking [18].

Due to a lack of knowledge or a system for the proper disposal of unused medicines, people in possession of those medicines may manage them in different ways. Rinsing of unwanted medicines down a sink, flushing them down the toilet, and throwing them in the trash were some of the commonest disposal methods commonly practiced [4, 8, 11, 19–24].

There were reports of trace amounts of medicines found in groundwater, surface bodies of water, and drinking water [25]. There were also many reports of adverse effects of these pharmaceuticals in other species [26]. A study conducted in southeast Queensland identified a significantly higher level of antibiotic and multiple antibiotic resistance in *E. coli* at point source sites in wastewater treatment plants (WWTPs). *Escherichia coli* resistant to ampicillin, tetracycline, sulfamethoxazole, and ciprofloxacin was identified [27]. These findings may be a result of postconsumption excretion as well as improper disposal of medicines.

Many of the developed countries have advanced disposal programs of unused medicines. In Australia, there has been the National Return and Disposal of Unwanted Medicines Project since 1998, fully supported by the government and pharmaceutical industry [10]. In the United Kingdom, pharmacy take-back programs are more abundant [28]. In the United States, medication disposal programs have been implemented by pharmacies and law enforcement agencies under the Secure and Responsible Drug Disposal Act of 2010 (DEA, 2014) [29]. In developing countries like those in Africa, unused medicines disposal management appears to be more problematic as reports from many countries (Mozambique, South Africa, Kenya, and Swaziland) indicate unsafe disposal practices characterized by unregulated, illegal, and indiscriminate disposal of unused medicines [30, 31]. Thus, in most developing countries, including Ethiopia, besides the high economic impact of waste generation by itself, medical waste management currently receives very little attention [32].

National survey data indicated that, in Ethiopia, the national average for availability of key essential drugs in health facilities was 70%, 85%, and 91% for public health facilities, regional drug stores, and private drug retail outlets, respectively. Public health facilities were the main sources of medicines (71%) followed by private pharmacies (18%) while the contribution of the informal sector as source of drugs was insignificant (<1%). The average number of drugs prescribed per encounter was also found to be 1.9, and the percentage of antibiotic use was 58% [33]. In countries like Ethiopia, where the majority (>80%) of the national medicines need is supplied by importation, medication wastage is therefore an additional burden.

Lack of uniform and nationwide guidance on how patients should safely dispose unused medications is also of great concern. The extent and problem of unused, expired, and unwanted medicines have not been fully studied in Ethiopia. As such, with due regard to the environmental, financial, and health-related consequences of unused medicines, the main purpose of this study was to determine the magnitude, types, and financial cost of unused medicines together with their disposal practice at households of Awi zone, Amhara regional state.

## 2. Methods

### 2.1. Study Design

A community-based cross-sectional study was conducted. A structured interviewer-administered questionnaire was used for data collection.

### 2.2. Study Area and Period

The study was conducted on households in Awi zone, Amhara regional state, Ethiopia. Awi zone is one of the 15 zones (including the 3 special (city) zones) found in the Amhara National Regional State. It is located at 700–2920 meters above sea level, and its land area is estimated at about 857,886 hectares. Based on the 2015 national census data and using a conversion factor, its population in 2018 is estimated to be around 1,264,203, of which 1,057,604 are rural residents and 206,599 are urban residents. Of the total population, 50.1% are male and 49.9% are female residents. The study was conducted from 23 April to May 22, 2018.

### 2.3. Sample Size Determination

The sample size was calculated using a single population proportion formula [34]. In the absence of previous prevalence data, it is advised to take 50% to get a maximum sample size, and thus the proportion of households with unused medicines was assumed to be 50% [34]. Considering the homogeneity of the population, taking a design effect of 1.2 and a 10% nonresponse rate, the final sample size was 507 households.

### 2.4. Sampling Techniques

A total of 23 kebeles (the smallest administrative unit in Ethiopia) (2 urban and 21 rural kebeles) from four woredas were selected using a multistage sampling technique. In this technique, the first step was taking a sample of woredas; thus, among the total 12 woredas found in Awi zone, 4 woredas were selected by taking 30% of the total woredas, and specific woredas were then selected by lottery method. Three rural woredas and one town administration were selected. Then, the number of kebeles to be sampled at each of the sampled woredas was determined by taking 30% of the total number of kebeles at each of those woredas. Specific kebeles were then selected using systematic random sampling technique using name of kebeles in alphabetic order as a sampling frame. The number of households to be sampled in each of the selected kebeles was determined using sample proportional to size method (by assigning the total sample size at that specific woreda to the total number of households at each of the selected kebeles), and specific households were selected using systematic random sampling technique by using house numbers from respective kebele offices as a sampling frame.

### 2.5. Data Collection Procedure

An adapted structured questionnaire was used after being modified to fit with the current study setup [9, 19, 35].

Seven health extension workers participated in the data collection. An adult member of a household who was well informed about the medicine taking behavior of the family was interviewed, and in situations where more than one such member was available, interviewees were selected randomly by using a lottery method.

The monetary value of medicines was determined by multiplying the unit cost of the medicines by the actual number of pills remaining in the container according to the list of drug prices provided by the Ethiopian Ministry of Health (EMOH). The monetary value of medicines includes only the price of medicines, not costs associated with handling the medicines or disposing of them or any other related costs.

### 2.6. Data Quality Assurance

Two-day training was provided for the data collectors. Pretest of the data collection instrument was conducted on 25 households prior to the actual data collection in order to check the applicability of the instrument and make necessary adjustments. These samples were not included in the final analysis. The collected data were checked for its completeness, consistency, and accuracy before entry to statistical software for analysis. The data were also cleaned for inconsistencies and missing values after entry to SPSS.

### 2.7. Data Processing and Analysis

The collected data were entered into Epi Data version 3.1 and then exported to SPSS version 21 for analysis. A chi-square test was conducted between different sociodemographic variables (independent variables) and the presence of unused medicine (the dependent variable). *P*-value of <0.05 was taken to declare significance at a 95% confidence interval. Predictors of unused medicines were identified using binary and multivariable logistic regression methods. Model fitness was checked by Nagelkerke's *R*^2^ test with the final model fit at *P*=0.215.

## 3. Results

### 3.1. Characteristics of the Respondents

A total of 507 households were included in the study. The majority of the study participants, 368 (72.6%), were female. The mean age of the study participants was 40 years, and the majority were between the ages of 30 and 65 (67.9%) (Table 1).

### 3.2. Prevalence of Unused Medicine

At the time of data collection, out of the total 507 households surveyed, 91 (17.9%) were found to have any kind of medicine at home. At the same time, 70 (13.8%) of the households among the total 507 were found to have unused medicines.

Among the total unused medicines found, 40 (44.4%) were expired at the time of the data collection period (between April 23 and May 22/2018), while the remaining 50 (55.6%) of the medicines were not expired and were usable.

Regarding the source of their medicines, 486 (95.9%) of the respondents reported that they collect their medicines from public health facilities (pharmacies), 13 (2.5%) of them from a friend, and 8 (1.6%) from other sources (using previously stored medicines instead of buying new). With regard to self-medication practices, 51 (10.1%) reported that they sometimes buy medicines by themselves without consulting their physician. The same number of study participants, 51 (10.1%), had also reported that they share their medicines with their family members and friends who are in need of those medicines.

More than two-thirds of the respondents, 343 (67.7%), obtain their medicines through out-of-pocket expenditure, while 151 (29.8%) reported that health insurance has covered their healthcare expenses including their medicines, and the remaining 13 (2.5%) obtained for free from their friends. More than half of the study participants 287 (56.6%) reported that current medicines are costly, while the remaining 220 (43.4%) thought that the prices are fair.

The majority of the study participants, 322 (63.5%), reported that they were informed about the medicines they took, while the remaining 185 (36.5%) reported that they were not informed about their medicines.

The chi-square result also showed that there was a statistically significant difference at *P*-value <0.05, in the presence of unused medications with regard to differences in monthly income, marital status, a view that medicines once purchased can be used with no time limit, medications sharing, and medicine awareness (Table 2).

The maximum number of unused medicines possessed by households was 5. Some households possessed more than one type of medicines and a total of 28 types of medicines in different dosage forms; 0.17 medicines per household were found (Table 3).

**Figure 1:**
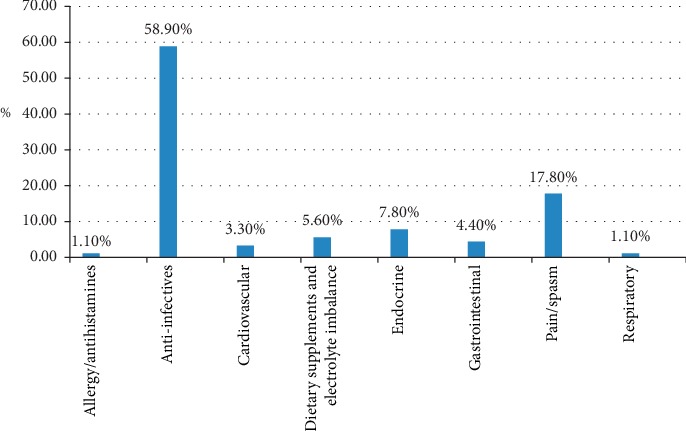
Types of unused medicines based on therapeutic categories at households in Awi zone, Amhara regional state, Ethiopia, 2018 *N* = 90.

Anti-infective medicines were found to be the most common unused medicines, 53 (58.9%), followed by antipain medicines, 16 (17.8%) (Figure 1).

The study participants were asked where they store medications they use (despite the availability of in-use medications at the time of data collection) and unused medications, and the majority of them had reported that they store both in-use and unused medicines in a shelf (*chegot*), 326 (64.3%) for in-use medicines and 37 (39.4%) for unused medicines. Some had reported more than one place for storage (Table 4).

### 3.3. Reasons for the Presence of Unused Medicines

People who pay for medications out of pocket by themselves were found 2.6 times more likely to have unused medications (AOR = 2.59, 95% CI of (1.30, 5.16)). People who did not have enough knowledge about medications (those who think medications once purchased can be used forever) were also found 4.8 times more likely to have unused medications (AOR = 4.87, 95% CI of (1.97, 12.02)). Similarly, People who did not have enough awareness about medications they took were found 3 times more likely to have unused medications (AOR = 3.04, 95% CI of (1.45, 6.38)) (Table 5).

Forgetfulness was the most common reason for the presence of unused medicines, 35 (33.7%), followed by discontinuation of medicines due to fast improvement, 30 (28.8%). Unused medicines due to expiry, 18 (17.3%); intolerance of medicine side effects, 11 (10.6%); change of treatment, 9 (8.6%); and medicines left unused due to death of a patient, 1 (0.9%), were the most common reasons reported by the study participants for the presence of unused medicines at home. There were also participants, 35 (6.9%), who thought that medicines once purchased can be used indefinitely, thus having no expiry date.

### 3.4. Disposal Practice for Unused Medicines

Regarding awareness about management of unused medicines and proper disposal methods, only 145 (28.6%) study participants out of the total 507 reported that they have previously heard about management practices of unused medicines and proper disposal methods, while the remaining 362 (71.4%) reported that they have never heard of them before. Among the 145 study participants with previous information, 32 (22%) obtained information through mass education programs and the remaining 113 (78%) through other sources (mass media, friends, etc.). However, none of the study participants had reported that they have ever experienced a medicine take-back event. The majority of the study participants, 441 (87%), stated that they were not informed about proper medicines disposal methods by their physician or pharmacist.

**Figure 2:**
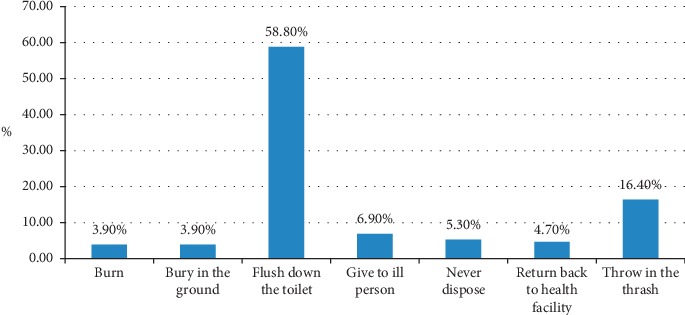
Disposal methods of unused medicines at households in Awi zone, Amhara regional state, Ethiopia, 2018 *N* = 70.

When asked about how they manage unused medicines, 38 (7.5%) of the study participants reported that they share those medicines with others who need them, 82 (16.2%) reported that they might use them in the future, and the remaining 387 (76.3%) reported that they discarded those unused medicines. Flushing unused medicines down the toilet was the most common disposal method, 298 (58.8%), followed by throwing medicines in the trash, 83 (16.4%) (Figure 2).

### 3.5. Monetary Value of Unused Medicines

The monetary value of unused medicines was determined for solid dosage forms (tablets and capsules) by multiplying the unit price of those medicines by the quantity left unused. A total of about 827ETB (29.5USD) worth of medicines, which is on average 1.6ETB (0.06USD) per household, was found unused (Table 6).

## 4. Discussion

The prevalence of unused medicines in the current study was found to be lower than that in a similar study conducted in Nigeria, in which 105 (80.8%) households had 635 medicines at their homes, 65.8% of which were unused medicines (3.2 unused medicines per household) [24]. This could be attributed to differences in access to pharmacies in the two study areas as well as differences in self-medication practices in Nigeria and Ethiopia, in which evidence showed that there is a higher self-medication practice in Nigeria as compared to Ethiopia [36, 37]. The fact that in Ethiopia public health facilities are the main sources of medicines (71%) can also indicate the probability of low self-medication practice, as medications to be dispensed on public health facilities are mainly prescription only medications [33].

In the current study, anti-infective and antipain medicines were the most common unused medicines in the community. Similar findings were reported from a study in Cairo, Egypt, in which antibiotics were the most common medicines returned unused, 20.15%, followed by medicines for gastrointestinal problems, 16.27% [7]. The greater abundance of antibiotics reflects the potential for the generation of this waste at households, the problem of lack of treatment adherence, the problem of antibiotic resistance, and the risk of self-medication and associated problems [38].

The most commonly reported reasons for the presence of unused medicines were forgetfulness, 33.7%, followed by fast symptom improvement, 28.8%. Similar findings with varying frequencies were found in studies conducted in Egypt and University of Gondar Specialized Teaching Hospital, Ethiopia [3, 7]. In the current study, the number of reasons for the presence of unused medicines reported by the participants was higher than the number of households that had unused medicines. This was due to the fact that a single household sometimes had more than one type of unused medicines at home and perhaps sometimes had the same medicine but purchased at different times, and the reasons for the nonutilization of those medicines were also sometimes different.

In the current study, people who pay for medicines out of pocket by themselves were found 2.6 times more likely to have unused medicines compared to those for whom health insurance has covered their medical expenses. This could be attributed to the fact that people who pay for medicines immediately out of pocket could tend to save their medicines so as to avoid further expenses for them as compared to those who utilized health insurance services as they may not realize/pay attention to the money they pay/save in the insurance services as it is gradual and not much at once.

People who did not have enough knowledge about medicines (those who think medicines once purchased can be used forever) were also found 4.8 times more likely to have unused medicines. This could be a factor as those people will tend to save those medicines for future consumption.

Similarly, people who did not have enough awareness about medicines they took were found to be 3 times more likely to discontinue their medicines and thus have unused medicines compared with those who had good awareness. This could be due to poor counseling services by health professionals/pharmacists during dispensing the medicines or due to some other reasons such as lack of awareness of the person who took the medicines.

Flushing of medicines down the toilet was the most common disposal method for unused medicines, 58.8%, followed by throwing them in the trash, 16.4%. Similar disposal practices were reported by studies conducted in Nigeria and Ghana [23, 24]. In another study conducted in Ghana, the study participants reported throwing unused medicines in a wastage bin (29%), burying them in the ground (38%), and flushing them down the toilet or sink (4%) as the most common disposal methods for unused medicines, whereas 21% of them reported sharing of those medicines with their families and relatives [39]. Although flushing down a sink/toilet and throwing into the trash were convenient ways for removing medicines from households given the absence of disposal instructions on the prescription drug labeling and medicine take-back programs in the country, these methods pose a significant environmental concern [10, 40].

### 4.1. Limitation of the Study

The cost of medicines was calculated only for solid dosage forms and parenteral medicines, due to the difficulty in determining the amount remaining for liquid preparations, and thus may not accurately indicate the actual cost of unused medicines. In addition, the cost was also calculated using the unit price of the medicines at the time of the study, and thus depending on when the medicines were purchased there may be a fluctuation in the unit price of the medicines due to the difference between the current price and that at the time of purchase.

## 5. Conclusion

Unused medicines were present in the community. Strategies aimed at educating the public regarding the general properties of medicines are needed, and guidelines on the safe disposal of unused medicines and an organized method of collection in the community need to be introduced. Drug take-back programs aimed at collecting and safely disposing of unused medicines in the community are also required. One option for this could be disposing of unused medicines from households together with the disposal program in the health facilities. In Ethiopia, disposal of unused and expired pharmaceuticals is conducted by a team of experts from the regulatory body (Ethiopian Food and Drug Administration (EFDA)), pharmacists, laboratorists, security officers (police), and other relevant stakeholders in a common disposal area using the disposal guideline provided by EFDA (formerly FMHACA (Food, Medicine, and Healthcare Administration and Control Authority of Ethiopia)) [41]. This could be achieved by placing drug collection sites at various areas in the community and educating the public to put unused medicines on the nearby collection site, with a disposal team conducting the collection, segregation, and steps related to disposal.

One other option for the collection of those unused medicines could be achieved by utilization of private firms involved in collection and disposal of other household wastes. In many areas of the country including the current study area, there are many privately organized firms (entrepreneurs) conducting such activities as a formal work for living. One barrier to this could be the knowledge gap and cautions to be taken on handling of drugs, as many of them may not have health-related background. However, this could be overcome by providing all the necessary training for handling of medicines upon collection and all the necessary cautions to be taken and/or by putting and managing them in a system. All of this needs regular financing, and the government together with donor agencies and pharmaceutical companies in the country's market could be a good source.

## Figures and Tables

**Table 1:** Characteristics of study participants at sampled *woreda*-*kebele*s in Awi zone, Amhara regional state, Ethiopia, 2018 *N* = 507.

Variables	Number	Percent
*Sex*		
Male	139	27.4
Female	368	72.6

*Age*		
18–30	147	29
31–40	127	25
41–50	144	28.4
51–60	60	11.8
>60	29	5.7

*Educational level*		
No formal education	264	52.1
Primary education	164	32.3
Secondary education	67	13.2
Higher education	12	2.4

*Monthly income (in ETB)*		
<500	193	38.1
500–1500	196	38.7
1501–2500	99	19.5
>2500	19	3.7

*Marital status*		
Single	32	6.3
Married	430	84.8
Divorced	27	5.3
Widowed	18	3.6

*Residency*		
Urban	58	11.4
Rural	449	88.6

*Family size*		
Less than 3	74	14.6
3–5	260	51.3
>6	173	34.1

**Table 2:** Chi-square test between having unused medicines at home and the independent variables *N* = 70.

Variables		Presence of unused medicines		Chi-square	*P*-value
		Yes (%)	No (%)		
Educational level	No formal education	36 (51.4)	228 (52.1)	1.999	0.573
	Primary education	24 (34.3)	140 (32)		
	Secondary education	7 (10.0)	60 (13.7)		
	Higher education	3 (4.3)	9 (2)		

Monthly income	<500	35 (50)	158 (36.2)	9.479	0.024
	500–1500	27 (38.6)	169 (38.7)		
	1501–2500	5 (7.1)	94 (21.5)		
	>2500	3 (4.3)	16 (43.2)		

Marital status	Single	3 (4.3)	29 (6.6)	10.413	0.015
	Married	53 (75.7)	377 (86.3)		
	Divorced	9 (12.9)	18 (4.1)		
	Widowed	5 (7.1)	13 (3)		

Residency	Urban	5 (7.1)	53 (12.1)	1.480	0.311
	Rural	65 (92.3)	384 (87.9)		

Source of medicines	Pharmacy	66 (94.3)	420 (96.1)	0.839	0.657
	Friends and other	4 (5.7)	17 (3.9)		

Source of payment	Health insurance	16 (22.9)	148 (33.9)	3.342	0.068
	Out of pocket	54 (77.1)	289 (66.1)		

Expensiveness of medicines	Yes	47 (67.1)	240 (54.9)	3.670	0.055
	No	23 (32.9)	197 (45)		

A view that medicines once purchased can be used with no time limit	Yes	18 (25.7)	17 (3.9)	44.715	0.001
	No	52 (74.3)	420 (96.1)		

Self-medication practice	Yes	6 (8.6)	45 (10.3)	0.199	0.831
	No	64 (91.4)	392 (89.7)		

Sharing of medicines	Yes	18 (25.7)	33 (7.6)	22	0.001
	No	52 (74.3)	404 (92.4)		

Awareness about medicines taken	Yes	57 (81.4)	265 (60.6)	11.251	0.001
	No	13 (18.6)	172 (39.4)		

Experiencing mass education about unused medicines management	Yes	3 (4.3)	29 (6.6)	0.564	0.601
	No	67 (95.7)	408 (93.3)		

**Table 3:** Types of unused medicines at households in sampled *woreda*-*kebele*s, Awi zone, Amhara regional state, Ethiopia, 2018, *N* = 90.

Name of medicine	Therapeutic category	Unit	Frequency	Percentage	Quantity left
Amoxicillin 125 mg/5 ml syrup	Antibiotic	Bottle	4	4.4	4
Amoxicillin 250 mg capsules	Antibiotic	Capsule	5	5.6	60
Amoxicillin 500 mg capsules	Antibiotic	Capsule	11	12.2	104
Amox + clavulanic acid 625 mg tablet	Antibiotic	Tablet	1	1.1	5
Ampicillin 500 mg capsules	Antibiotic	Capsule	1	1.1	2
Azithromycin 250 mg tablet	Antibiotic	Tablet	1	1.1	4
Berantin cough	Antitussive	Bottle	1	1.1	1
Ceftriaxone 1 g injection	Antibiotic	Vial	1	1.1	1
Chloramphenicol 250 mg capsules	Antibiotic	Capsule	1	1.1	8
Ciprofloxacin 500 mg tablet	Antibiotic	Tablet	3	3.3	30
Clobetasol propionate	Corticosteroid	Tube	1	1.1	3
Cotrimoxazole 240 mg/5 ml oral susp.	Antibiotic	Bottle	4	4.4	4
Cotrimoxazole 480 mg tablet	Antibiotic	Bottle	1	1.1	10
Coartem 140 mg tablet	Antimalarial	Tablet	5	5.6	24
Doxycycline 100 mg capsules	Antibiotic	Capsule	4	4.4	40
Hydrochlorothiazide 25 mg tablet	Diuretic	Tablet	1	1.1	10
Ibuprofen 400 mg tablet	Antipain	Tablet	1	1.1	10
Indomethacin 25 mg tablet	Antipain	Tablet	1	1.1	9
Iron tablet	Nutritional supp.	Tablet	1	1.1	3
Iron with folic acid tablet	Nutritional supp.	Tablet	4	4.4	108
Methyldopa 250 mg tablet	Antihypertensive	Tablet	1	1.1	20
Metronidazole 250 mg capsules	Antibiotic	Capsule	5	5.6	52
Metronidazole 125 mg/5 ml bottle	Antibiotic	Bottle	1	1.1	1
Norfloxacin 400 mg tablet	Antibiotic	Tablet	1	1.1	13
Omeprazole 20 mg capsule	Antigastric ulcer	Capsule	4	4.4	91
Microgynon/levonorgestrel/0.15 mg tab.	Contraceptive	Tablet	1	1.1	28
Oral rehydration salts (ORS)	Nutritional supp.	Sachet	2	2.2	2
Paracetamol 100 mg tablet	Antipain	Tablet	1	1.1	6
Paracetamol 500 mg tablets	Antipain	Tablet	12	13.3	100
Spironolactone 25 mg tablet	Diuretic	Tablet	1	1.1	10
Tramadol 50 mg capsule	Antipain	Capsule	2	2.2	48
TTC eye ointment	Antibiotic	Tube	3	3.3	4
TTC HCl cream	Antibiotic	Tube	1	1.1	1
Zinc sulphate 20 mg tablet	Nutritional supp.	Tablet	3	3.3	20
Total			90	100.0	836

**Table 4:** Storage conditions of in-use and unused medicines at households in sampled *woreda*-*kebele*s in Awi zone, Amhara regional state, Ethiopia, 2018 *N* = 507.

Storage place	In-use medications		Unused medications		Chi-square	*P*-value
	Frequency	Percent (%)	Frequency	Percent (%)		
Shelf (chegot)	326	64.3	37	7.3	71.021	0.003
Box	147	29.0	36	7.1		
Pocket	10	2.0	5	1.0		
Bag	5	1.0	4	0.8		
Basket (agelgil)	10	2.0	9	1.8		
Cupboard	6	1.2	3	0.6		
Other (under a pillow)	3	0.6	0	0		
Not storing	0	0	413	81.5		
Total	507	100	507	100		

**Table 5:** Predictors for the prevalence of unused medicines at sampled *woreda*-*kebele*s, Awi zone, Amhara regional state, Ethiopia, 2018 *N* = 507.

Variables	Percentage of householders	Crude odds ratio	*P*-(<0.25) (COR)	Adjusted odds ratio	*P*-(<0.05) (AOR)
*Monthly income* (*ETB*)					
1501–2500	99 (19.5%)	3.52 (0.76,16.2)	0.106	3.82 (0.78, 18.74)	0.098
>2500	19 (3.7%)				

*Marital status*					
Single	32 (6.3%)	3.71 (0.77, 17.93)	0.102	2.03 (0.32, 12.61)	0.445
Married	430 (84.8%)	2.73 (0.93, 7.98)	0.065	1.87 (0.52, 6.66)	0.331
Widowed	18 (3.6%)				

*Residence*					
Urban	58 (11.4%)				
Rural	449 (88.6%)	1.79 (0.69, 4.65)	0.230	0.83 (0.25, 2.70)	0.76

*Source of payment for medicines*					
Health insurance	151 (29.8%)				
Out of pocket	343 (67.7%)	1.72 (0.95, 3.12)	0.070	**2.59 (1.30, 5.16)**	0.006

*Feeling medicines are expensive*					
Yes	287 (56.6%)	1.67 (0.98,2.85)	0.057	1.85 (0.99, 3.48)	0.053
No	220 (43.4%)				

*Thinking medicines have no expiry date* (*they can be used forever*)					
Yes	35 (6.9%)	8.55 (4.15, 17.61)	0.0000001	**4.87 (1.97, 12.02)**	0.001
No	472 (93.1%)				

*Sharing of medicines with others*					
Yes	51 (10.1%)	4.23 (2.22, 8.05)	0.00001	1.49 (0.65, 3.40)	0.339
No	456 (89.9%)				

*Awareness about medicines taken*					
Yes	322 (63.5%)				
No	185 (36.5%)	2.84 (1.51, 5.35)	0.001	**3.04 (1.45, 6.38)**	0.003

**Table 6:** Cost of unused medicines at households in sampled *woreda*-*kebele*s in Awi zone, Amhara regional state, Ethiopia, 2018 *N* = 34.

Name of medicine	Unit	Therapeutic category	Quantity left	Unit price	Total price
Amoxicillin 125 mg/5 ml syrup	Bottle	Antibiotic	4	—	—
Amoxicillin 250 mg capsules	Capsule	Antibiotic	60	0.5	30
Amoxicillin 500 mg capsules	Capsule	Antibiotic	104	1.03	197.12
Amox + clavulanic acid 625 mg tablet	Tablet	Antibiotic	5	3.2	16
Ampicillin 500 mg capsules	Capsule	Antibiotic	2	1	2
Azithromycin 250 mg tablet	Tablet	Antibiotic	4	4.6	18.4
Berantin cough	Vial	Antitussive	1	—	—
Ceftriaxone 1 g injection	Vial	Antibiotic	1	—	—
Chloramphenicol 250 mg capsules	Capsule	Antibiotic	8	0.85	6.8
Ciprofloxacin 500 mg tablet	Tablet	Antibiotic	30	1.03	30.9
Clobetasol propionate	Tube	Corticosteroid	3	—	—
Cotrimoxazole 240 mg/5 ml oral susp.	Bottle	Antibiotic	4	—	—
Cotrimoxazole 480 mg tablet	Bottle	Antibiotic	10	0.4	4
Coartem 140 mg tablet	Tablet	Antimalarial	24	1.11	26.64
Doxycycline 100 mg capsules	Capsule	Antibiotic	40	0.86	34.4
Hydrochlorothiazide 25 mg tablet	Tablet	Diuretic	10	0.5	5
Ibuprofen 400 mg tablet	Tablet	Antipain	10	0.45	4.5
Indomethacin 25 mg tablet	Tablet	Antipain	9	0.41	3.69
Iron tablet	Tablet	Nutritional supp.	3	0.55	1.65
Iron with folic acid tablet	Tablet	Nutritional supp.	108	0.62	66.96
Methyldopa 250 mg tablet	Tablet	Antihypertensive	20	0.96	19.2
Metronidazole 250 mg capsules	Capsule	Antibiotic	52	0.33	17.16
Metronidazole 125 mg/5 ml bottle	Bottle	Antibiotic	1	—	—
Norfloxacin 400 mg tablet	Tablet	Antibiotic	13	1.12	14.56
Omeprazole 20 mg capsule	Capsule	Antigastric ulcer	91	0.46	41.86
Microgynon/levonorgestrel/0.15 mg tab.	Tablet	Contraceptive	28	6.9	193.2
Oral rehydration salts (ORS)	Sachet	Nutritional supp.	2	8.95	17.9
Paracetamol 100 mg tablet	Tablet	Antipain	6	0.13	0.78
Paracetamol 500 mg tablets	Tablet	Antipain	100	0.2	20
Spironolactone 25 mg tablet	Tablet	Diuretic	10	1.03	10.3
Tramadol 50 mg capsule	Capsule	Antipain	48	0.91	43.68
TTC eye ointment	Tube	Antibiotic	4	—	—
TTC HCl cream	Tube	Antibiotic	1	—	—
Zinc sulphate 20 mg tablet	Tablet	Nutritional supp.	20	Free	—
Total			836		**826.7ETB**

## Data Availability

The datasets generated and/or analyzed during the current study are not available in public due to the requirement of confidentiality, upon which the study was approved by the Institutional Review Board and consent was secured from the study participants, but are available from the corresponding author on reasonable request.

## Supplementary Materials

Supplementary Materials Part I: sociodemographic data. Part II: medicine management related data. Part III: visual inspection of unused medicines.
