# Supplementary material for: Prevalence and Predictors of Storage of Unused Medicines among Households in Northwestern Ethiopia
Source: J Environ Public Health. 2020 Mar 26;2020:8703208. doi: 10.1155/2020/8703208 (PMC7136802; doi:10.1155/2020/8703208)
Supplement: Supplementary Materials — Part I: sociodemographic data. Part II: medicine management related data. Part III: visual inspection of unused medicines. [file 8703208.f1.pdf]

Appendix

Questionnaire

Part I: Socio-demographic data

1. Sex
   A. Male　　B. Female
2. Age
   A. 10-17　　C. 30-65
   B. 18-29　　D. >65
3. Educational level
   A. No education
   B. Primary education (Grade 1-8)
   C. Secondary education (Grade 9-12)
   D. Higher education (College/ University)
4. Monthly income
   A. <500　　C. 1501-3000
   B. 500-1500　　D. >3000
5. Marital status
   A. Single　　C. Divorced
   B. Married　　D. Widowed
6. Residency
   A. Urban　　B. Rural
7. Family size? _______________

Part II: Medicine management related data

1. Where did you get your Medicines?
   A. Pharmacy
   B. From a friend
   C. Other (specify)…
2. Who pays for the Medicines you buy?
   A. Health insurance

B. Out of pocket (yourself)

C. Other…

3. Where do you store Medicines at home?

   3.1 In-use Medicines___________________________________

   3.2 Unused Medicines__________________________________

4. Do you have in-use Medicines at home?

   A. Yes B. No

5. Do you have unused Medicines at home?

   A. Yes B. No

6. Do you think Medicines are expensive?

   A. Yes B. No

7. Do you think Medicines once purchased can be used indefinitely in the future?

   A. Yes B. No

8. Do you sometimes buy medicines by your-selves without consulting your medical practitioner?

   A. Yes B. No

9. Are there any unused Medicines at your home that were left because of you forgot them to take or relieved before completion?

   A. Yes B. No

10. Are there unused Medicines at your home that were left because of the change of treatment?

    A. Yes B. No

11. Are there unused Medicines at your home that were left due to intolerable side effects?

    A. Yes B. No

12. Are there unused Medicines at your home that were left due to passed expiry date before you finish them?

    A. Yes B. No

13. Are there unused Medicines at your home that you left taking them because of your conditions were improved?

    A. Yes B. No

14. Are there unused Medicines at your home that were left unused because someone died while on Medicines before finishing them?

    A. Yes   B. No

15. What do you do with unused Medicines?

    A. Share to others   C. Discard/dispose of them

    B. Use in the future   D. Other (specify)……

16. If someone at your home/ friend is sick and asks you for your Medicines, will you share them?

    A. Yes   B. No

17. Were you aware of the Medicines you took? (How to take, how much to take, when to take…)

    A. Yes   B. No

18. Were you informed by your medical practitioner/ pharmacist about the disposal methods of unused Medicines?

    A. Yes   B. No

19. Have you ever heard of proper medicine disposal methods?

    A. Yes   B. No

20. How do you dispose of unused medicines at your home?

    A. Throw to the trash
    B. Burn unused Medicines
    C. Bury in the ground
    D. Flush down the toilet
    E. Return unused Medicines to hospital/ pharmacy
    F. Give to the ill person
    G. Never dispose of unused medicines

21. Did you ever experience a Medicine take-back event in your residency?

    A. Yes   B. No

22. Have you ever participated in a mass education program about unused Medicine management in your residency?

    A. Yes
    B. No

Part III: Visual inspection of unused Medicines

1. Number of Medicines unused (based on type)? ____________________
2. Name of Medicines unused? ____
3. Type of Medicine based on therapeutic class?

   ____

A. Allergy (antihistamines)
B. Cardiovascular
C. Dietary (related to electrolyte or vitamin imbalances)
D. Endocrine (e.g., diabetes, hypothyroidism, reproductive hormone replacement)
E. Gastrointestinal
F. Immunologic (e.g., cancer, rheumatoid arthritis, immunosuppression)
G. Infectious disease
H. Mental health (e.g., depression, bipolar disorder, schizophrenia, anxiety, attention deficit disorder, insomnia, seizure disorder)
I. Nausea
J. Pain/spasm
K. Respiratory
L. Geriatric/miscellaneous (e.g., urinary incontinence, osteoporosis, dementia, Parkinson's disease, benign prostatic hyperplasia, erectile dysfunction

4. Quantity left unused (for each type of Medicine)? ______________________________

   ____

5. Date of expiry (for each type of Medicine)?

   ____

   ____

6. Unit price/ Price per pack (for each type of Medicine), based on average retail price?

   ____
